# Sleep and non-motor symptoms in Parkinson’s disease

**DOI:** 10.1007/s00702-013-0966-4

**Published:** 2013-01-22

**Authors:** Antonia Maass, Heinz Reichmann

**Affiliations:** Department of Neurology, University Hospital ‘Carl Gustav Carus’, University of Technology Dresden, Fetscherstrasse 74, 01307 Dresden, Germany

**Keywords:** Parkinson’s disease, Non-motor symptoms, Sleep disorders, Quality of life

## Abstract

Beyond the cardinal motor symptoms, bradykinesia, rigidity, tremor and postural instability, defining the diagnosis of Parkinson’s disease, there is a big spectrum of non-motor features that patients may suffer from and that may reduce their quality of life. Non-motor symptoms are not only frequent but also often under-reported by patients and caregivers. As they are frequently under-recognized by clinicians, they remain consequently under-treated. This review wants to give a short overview of the importance of non-motor symptoms on patients’ quality of life and helpful assessment tools that might facilitate recognition of non-motor features during clinical setting. Given the wide range of non-motor symptoms in Parkinson’s disease, we concentrate on common issues such as depression and sleep disorders like sleep-onset insomnia or sleep maintenance insomnia and restless legs syndrome. Thereby, we present some recent studies that have investigated the efficacy of dopaminergic drugs, especially dopamine agonists, revealing possible treatment strategies and thus improving disease management.

## Introduction

Parkinson’s disease (PD) is a neurodegenerative disorder accompanied by both motor and non-motor symptoms (NMS). Non-motor features can be present at any stage and may precede the onset of motor signs (Chaudhuri et al. 2005; Chaudhuri and Naidu 2008). NMS comprise symptoms such as hyposmia, autonomic dysfunction, gastrointestinal and sensory problems, neuropsychiatric symptoms and sleep disorders (Sommer et al. 2004; Haehner et al. 2007; Ziemssen and Reichmann 2007; Chaudhuri and Schapira 2009). The importance of NMS lies in the fact that they are not only frequent but also have a great impact on patients’ quality of life. Beyond immobility or slowness, non-motor features account for the most common complaints of PD patients (Lee et al. 2007). Beneath motor fluctuations in advance PD, the most troublesome problems are ‘non-motor’-like mood changes, drooling and sleep problems (Politis et al. 2010). From the patients’ point of view not only the reduction of slowness or walking difficulties, but also fatigue, contributes to a successful treatment outcome (Nisenzon et al. 2011). Concerning the frequency of NMS, the PRIAMO study revealed that about 98 % of patients suffer from NMS. Thereby symptoms like apathy, fatigue, psychiatric issues as well as deficits in attention and memory had the most negative impact on quality of life (Barone et al. 2009). In line with that, several studies revealed that psychosocial factors like depression accounted significantly for quality of life (Global Parkinson’s Disease Survey Steering Committee 2002; Schrag et al. 2000). Depending on the study, the mean number of NMS lies between 7 and 12 (Chaudhuri et al. 2006; Martinez-Martin et al. 2007; Barone et al. 2009). Unfortunately, if not specifically requested, NMS are often under-reported and poorly recognized thus remaining under-treated (Chaudhuri et al. 2010).

## Recognition and assessment of NMS

**Fig. 1:**
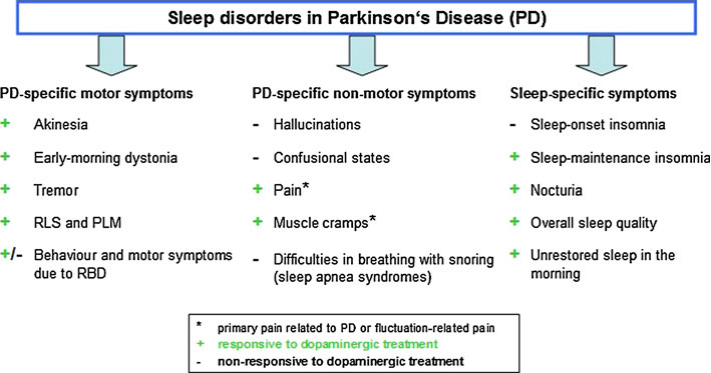
Different aspects of sleep disturbances in PD outlined in the PDSS-2 and their response to dopaminergic treatment (modified from Chaudhuri and Schapira 2009; Trenkwalder et al. 2011b). *PLM* periodic limb movements

Given the frequency as well as the impact of NMS on PD patients’ quality of life and the consequent implications on therapeutic strategies, clinicians need to consider these issues in their patient interviews. To date, there are some helpful tools facilitating recognition and assessment of NMS. For example, NMSQuest is an internationally used and validated questionnaire enabling rapid screening of possible non-motor features (Chaudhuri et al. 2006). As sleep disturbances, among other NMS, have been reported in 60–90 % and are unrecognized in over 40 % of patients with PD, clinicians should actively and routinely inquire about sleep patterns during consultation (Trenkwalder 1998; Korczyn 2006; Chaudhuri et al. 2010). The Parkinson Disease Sleep Scale (PDSS) and its revised version PDSS-2 represent a useful instrument specifically developed to assess specific aspects of sleep disturbances and their severity. Problems of sleep in PD consist of sleep fragmentation with increased periods of wakefulness during the night, nocturnal akinesia with or without morning dystonia, rapid eye movement sleep behaviour disorder (RBD), restless legs syndrome (RLS), hallucinations and other neuropsychiatric symptoms, sleep apnea syndromes and nocturia (Trenkwalder et al. 2011b; Fig. 1). The revised PDSS-2 meets these nighttime features of PD patients in large part, reflecting a greater spectrum of nocturnal problems than the original PDSS. Further, the PDSS-2 is easy to handle for patient or caregiver and shows high validity and reliability (Trenkwalder et al. 2011b).

## NMS—dopaminergic dysfunction and efficacy of dopaminergic drugs

Dysfunction of dopaminergic pathways evidently contributes to a range of non-motor symptoms in PD thus highlighting the role of dopaminergic treatments to improve certain aspects of NMS (Wolters and Braak 2006; Fig. 1). To date, there are several clinical trials investigating the efficacy of dopaminergic drugs on key NMS such as depression, RLS and sleep disturbances.

### Depression

Against the background of dopaminergic dysfunction contributing to the pathophysiology of depression, several studies highlighted the antidepressant role of dopamine agonists both in major depression and depression in PD (Rektorova et al. 2003; Lemke et al. 2006; Dunlop and Nemeroff, 2007). Comparing pramipexole and pergolide in PD patients with mild or moderate depression as add-on therapy to levodopa, both substances showed significantly reduced depression scores (Rektorova et al. 2003). Raising the question whether the antidepressant benefit of pramipexole only represented treatment-related motor improvement, Barone et al. compared pramipexole with sertraline, a conventional antidepressant, in PD patients with depression. Depression scores decreased in both treatment groups, but a significantly greater proportion of patients recovered under pramipexole compared with sertraline (Barone et al. 2006). The antidepressant effects of pramipexole have further been under-scored in a placebo-controlled trial. Beck depression inventory (BDI) score as primary and UPDRS motor score as secondary endpoint significantly decreased in the pramipexole group. Thereby, the direct effect of pramipexole on depressive symptoms accounted for 80 % of total treatment effect thus indicating a ‘true’ antidepressant effect (Barone et al. 2010). Ropinirole, another dopamine agonist, may as well have a mood-regulating action. The EASE-PD study assessed the antiparkinsonian efficacy of prolonged release ropinirole in advanced cases of PD as adjunct therapy to levodopa. Compared with placebo, ropinirole showed significant changes in BDI suggesting an antidepressant action. Nevertheless, confounding effects of motor improvement need to be considered (Pahwa et al. 2007).

### Sleep disorders

With dopamine playing a complex role in the sleep-wake cycle, some sleep disturbances in PD might be dopamine-sensitive (Rye and Jankovic 2002). In PD, common sleep problems are difficulties with falling asleep (sleep-onset insomnia) and staying in sleep (sleep-maintenance insomnia), respectively. Sleep-maintenance insomnia is probably associated with a range of problems such as nocturnal akinesia and related motor and non-motor symptoms (e.g. RLS, RBD, nocturia; Fig. 1) (Lees et al. 1988). There is evidence that levodopa at bedtime and in a prolonged release formulation, respectively, lead to an improvement in sleep quality as well as nocturnal and morning motor state (Leeman et al. 1987; Van den Kerchove et al. 1993; Stocchi et al. 1998). The CLEOPATRA-PD study compared the efficacy of oral pramipexole and transdermal rotigotine in advanced-stage PD with placebo. Both dopamine agonists showed similar and significant efficacy in reduction of absolute off-time as primary outcome measure and significant improvement in secondary efficacy variables such as the PDSS (Poewe et al. 2007). Similar results were reported in the EASE-PD study with 24-h prolonged release ropinirole as adjunct therapy to levodopa (Pahwa et al. 2007). Specifically focusing on nocturnal disabilities and early morning akinesia in PD patients, the recent RECOVER study investigated the effects of transdermal rotigotine. In this multinational, double-blind trial, 287 PD patients were either randomized to receive placebo (80 completed) or rotigotine (166 completed). Over 1–8 weeks rotigotine was titrated to an optimal dose and maintained for a 4-week period. Concerning primary outcome measures, rotigotine lead to significantly greater improvements in UPDRS Part III scores in early morning and PDSS-2 total scores. Trenkwalder et al. concluded that 24-h delivery of rotigotine lead to an improvement of sleep maintenance insomnia by reducing nocturnal PD-specific symptoms (Trenkwalder et al. 2011a). Another open-label study, as well investigating the efficacy of transdermal rotigotine, found an overall improvement in early morning motor performance, nocturnal akinesia, dystonia, cramps, nocturia and overall sleep quality (Giladi 2010). In concordance with these findings, several open-label studies suggested that long-acting dopaminergic agents ameliorate sleep maintenance in PD patients, e.g. via long-acting cabergoline or continuous jejunal levodopa (Romigi et al. 2006; Honig et al. 2009). Beneath the alleviating effect of dopamine agonists on some sleep problems in PD, the clinical practitioner always has to keep in mind increased daytime sleepiness and sudden onset of sleep as possible adverse events when using specific dopamine agonists such as pramipexole or ropinirole (Frucht et al. 1999; Dhawan et al. 2006; Gjerstad et al. 2006). In case of occurrence, withdrawal or dose reduction of the dopaminergic agent needs to be considered.

RBD is a parasomnia characterised by dream-enacting behaviours related to vivid, usually frightening dreams and loss of muscle atonia during REM sleep and may predate the diagnosis of PD. Besides clonazepam as the drug of choice for RBD (Aurora et al. 2010), melatonin has been shown to be useful in a small, double-blind, placebo-controlled trial (Kunz and Mahlberg 2010). Pramipexole may also be considered although there is contradictory evidence revealing either beneficial or lacking effect on RBD (Fantini et al. 2003; Kumru et al. 2008). Concerning RLS, the European RLS Study Group provides a comprehensive therapeutic algorithm recommending levodopa, dopamine agonists, gabapentin and other agents depending on the clinical manifestation and aetiology (Garcia-Borreguero et al. 2012).

## Conclusion

NMS in PD are common and occur across all stages of the disease. Though representing a key determinant of quality of life, NMS are often under-reported by patients and under-recognized by health practitioners consequently remaining untreated. To overcome this dilemma, helpful patient-centred questionnaires have been developed alleviating the recognition and assessment of NMS, e.g. the Parkinson Disease Sleep Scale comprising a big spectrum of nocturnal disturbances in PD. Against the background of dopaminergic dysfunction accounting for a range NMS, there is evidence of effective dopaminergic treatment. Dopamine agonists improve several NMS e.g. reducing sleep problems and thus potentially improving quality of life.
